# Novel murine model reveals an early role for pertussis toxin in disrupting neonatal immunity to *Bordetella pertussis*

**DOI:** 10.3389/fimmu.2023.1125794

**Published:** 2023-02-08

**Authors:** Colleen J. Sedney, Amanda Caulfield, Kaylan K. Dewan, Uriel Blas-Machado, Maiya Callender, Nancy R. Manley, Eric T. Harvill

**Affiliations:** ^1^ Department of Infectious Diseases, College of Veterinary Medicine, University of Georgia, Athens, GA, United States; ^2^ Department of Pathology, College of Veterinary Medicine, University of Georgia, Athens, GA, United States; ^3^ Department of Genetics, Franklin College of Arts and Sciences, University of Georgia, Athens, GA, United States

**Keywords:** neonatal, pertussis (whooping cough), pertussis toxin, T cells, immunity

## Abstract

The increased susceptibility of neonates to specific pathogens has previously been attributed to an underdeveloped immune system. More recent data suggest neonates have effective protection against most pathogens but are particularly susceptible to those that target immune functions specific to neonates. *Bordetella pertussis* (*Bp*), the causative agent of “whooping cough”, causes more serious disease in infants attributed to its production of pertussis toxin (PTx), although the neonate-specific immune functions it targets remain unknown. Problematically, the rapid development of adult immunity in mice has confounded our ability to study interactions of the neonatal immune system and its components, such as virtual memory T cells which are prominent prior to the maturation of the thymus. Here, we examine the rapid change in susceptibility of young mice and define a period from five- to eight-days-old during which mice are much more susceptible to *Bp* than mice even a couple days older. These more narrowly defined “neonatal” mice display significantly increased susceptibility to wild type *Bp* but very rapidly and effectively respond to and control *Bp* lacking PTx, more rapidly even than adult mice. Thus, PTx efficiently blocks some very effective form(s) of neonatal protective immunity, potentially providing a tool to better understand the neonatal immune system. The rapid clearance of the PTx mutant correlates with the early accumulation of neutrophils and T cells and suggests a role for PTx in disrupting their accumulation. These results demonstrate a striking age-dependent response to *Bp*, define an early age of extreme susceptibility to *Bp*, and demonstrate that the neonatal response can be more efficient than the adult response in eliminating bacteria from the lungs, but these neonatal functions are substantially blocked by PTx. This refined definition of “neonatal” mice may be useful in the study of other pathogens that primarily infect neonates, and PTx may prove a particularly valuable tool for probing the poorly understood neonatal immune system.

## Introduction

Newborns and young children are highly susceptible to some infectious diseases, such as measles, respiratory syncytial virus (RSV), and whooping cough (1–3). This has been attributed to aspects of fetal-maternal tolerance and/or an “immature” immune system which is believed to generate weaker, less inflammatory responses relative to adults (4–6). However, neonates are not extraordinarily vulnerable to all infections, indicating that infants and young children are capable of mounting very effective, protective immune responses against most pathogens, even prior to maturation of the thymus.

The classical adult immune system that is most well-known and studied is comprised of adult T cells (T_adult_) which develop from bone marrow hematopoietic stem cells and mature in a fully differentiated thymus. These T_adult_ undergo a complex selection process to generate antigen-specific immune responses to many pathogens (7). Prior to the establishment of the classical adult immune system, neonates are replete with distinct populations of immune cells, including myeloid-deriver suppressor cells, CD71+ erythroid cells, and neonatal T cells (6, 8–12). Prominent amongst these fetal liver hematopoietic stem cell-derived T cells are those that display a virtual memory phenotype (T_VM_) (CD3^+^CD44^hi^CD49d^lo^CXCR3^+^Eomes^+^) and have a broadly reactive T cell receptor (9, 10, 13, 14). Importantly, these cells generate rapid and robust responses to early infection with various pathogens, which differs greatly from the typical delay in adaptive immune responses facilitated by T_adult_ (5). Recent studies have demonstrated that neonatal T cells can effectively expand and respond to viral and bacterial infections (15–18). While T_VM_ and other neonatal T cell subsets are prominent in neonates, most are gradually outnumbered and displaced by T_adult_ derived from bone marrow-derived progenitors matured in the thymus during the transition from neonate to adult.

Our ability to study neonatal immunity using the mouse model has been confounded by the very rapid thymic maturation in mice that begins to generate adult immune cells in the first week after birth. Due to this, experiments that involve mice older than one week (7 days) cannot clearly distinguish the effects of neonatal and adult T cells (9, 19–22). This is in contrast to human children, in whom thymic development is much slower, providing a wider window of vulnerability to some pathogens that appear to target this particular stage. One such pathogen is *Bordetella pertussis* (*Bp*), the etiologic agent of whooping cough, an internationally recognized re-emerging infectious disease that is highly virulent in neonates. It is estimated that there are over 5.1 million cases of whooping cough in children under 1-year-old annually, with nearly 86,000 of these cases resulting in infant mortality (3). Additionally, infection with *Bp* is associated with a number of complications in neonates and young children, including pneumonia, seizures, pulmonary hypertension, and encephalopathy (23). Extensive work has greatly informed our understanding of how this pathogen interacts with the adult immune system, leading to the development of vaccines capable of preventing disease in children, adolescents, and adults. However, due to the limitations of available models, our understanding of the neonatal response to *Bp* has resulted in few preventative options in the very young. Thus, extraordinary lengths are often taken to prevent infant exposure, for example by booster vaccinating all their potential contacts, a practice referred to as “cocooning” (20, 21, 24)

One of the major virulence factors that contributes to pertussis disease is pertussis toxin (PTx), an AB_5_ toxin that disrupts G protein coupled receptor signaling in various cell types (25, 26). PTx also interacts with T cell receptors to initiate signaling events and causes desensitization to signals such as chemokines (27, 28). PTx has also been demonstrated to inhibit neutrophil recruitment in early infection, resulting in delayed control of *Bp* (29). One of the most notable published effects of PTx on young mice was observed with animals that were challenged with *Bp* at 7-days-old (P7) then evaluated 7 days later in mice that were 14-days-old (P14), which suggested a special interaction between PTx and the “neonatal” murine immune system (22). However, the rapid development of the murine thymus begins well before P14, so the relative contributions of neonatal and adult-like immune cells are difficult to distinguish in this experimental setup.

Here we demonstrate that five- to eight-day-old mice (P5-P8) have substantial numbers of T_VM_ in the lungs during the first week of life, but by ten to fourteen-days-old (P10-P14), T_VM_ numbers in the lungs are already largely eclipsed by numbers of T_adult_. To more clearly separate the interactions of *Bp* with neonatal immunity, we present a novel model that focuses on the time period before the introduction of substantial T_adult_. We demonstrate significantly increased sensitivity to *Bp* growth and expansion in P5 mice, relative to mice even two days older (P7). Although highly susceptible to wild type (WT) *Bp*, P5 mice rapidly controlled and eliminated a PTx-deficient mutant of *Bp.* Efficient neonatal immune-mediated clearance was associated with rapid accumulation of neutrophils within 2 hours post inoculation and T cells within 1 day post inoculation. This experimental system more completely focuses on neonatal immunity, demonstrating that it can be highly effective against respiratory infections. Importantly, our data indicate that PTx specifically disrupts these neonatal-specific functions, potentially explaining the extraordinary sensitivity of newborns to *Bp* and not all other pathogens.

## Materials and methods

### Bacterial strains and growth

The *B. pertussis* strains Tohama 1 (WT *B. pertussis*) and BPH101, an isogenic pertussis toxin-deficient derivative (*B. pertussisΔptx*) have been previously described (22, 30–32). Bacteria were maintained on Bordet-Gengou agar (Difco) supplemented with 10% defibrinated sheep blood (Hema Laboratories). Liquid cultures were grown overnight in Stainer-Scholte broth at 37°C to mid-log phase then maintained in 20% glycerol stocks at -80°C for use as inoculum. Purified pertussis toxin (PTx) was obtained from Sigma-Aldrich as a lyophilized powder and resuspended with 500 μl PBS (P7208-5OUG). The purified PTx was added to the described inoculum at a concentration of 10 ng/μl.

### Mouse experiments

Six- to eight-week old female and male C57BL/6J (00664) mice were procured from the Jackson Laboratory (Bar Harbour, ME) and bred in the Harvill mouse colony (University of Georgia, GA). All mice were maintained in specific pathogen-free facilities, and all experiments were conducted following institutional guidelines. Pups were utilized at the indicated ages (five-, seven-, eleven-, fourteen-, twenty-five-, and twenty-eight-days-old) and six- to eight-week old mice were utilized for adult experiments. Mice were lightly sedated with 5% isoflurane (Pivetal) and inoculated (10^4^ CFU suspended in either 15 μl PBS for neonates and 50 μl PBS for adults) by pipetting the inoculum as droplets into their external nares to be inhaled. At the indicated timepoints mice were euthanized *via* CO_2_ inhalation and/or decapitation. Organs were excised and homogenized in 1 ml PBS, serially diluted, and plated on BG agar to quantify bacterial numbers. Colonies were counted following incubation for five days at 37°C.

### Flow cytometry

Lungs were processed and stained as previously described (33). Viable cells were identified with Zombie Aqua (Biolegend). 0.35 μl of each extracellular antibody was added to each sample. Antibodies to identify T and B cell populations included anti-CD45 AF700 (clone: 30-F11, Biolegend), anti-CD3 APC (clone:17A2, Biolegend), anti-CD4 VF450 (clone:RM4-5, Tondo Biosciences), anti-CD8 APC Fire 750 (clone:53-6.7, Biolegend), and anti-CD19 PerCP/Cy5.5 (clone:1D3/CD19, Biolegend). Antibodies to identify virtual memory T cells (T_VM_) and naïve adult T cells (CD44^lo^CD49d^lo^) (34) included anti-CD8 BV650 (clone: 53-6.7, BD Biosciences), anti-CD4 AF700 (clone:RM4-55, Biolegend), anti-CD44 PE/Cy7 (clone: IM7, Biolegend), anti-CD49d APC Fire 750 (clone:R1-2, Biolegend), anti-CD3 FITC (clone: 17A2, Biolegend), anti-TCRg/d PE/Cy5 (clone: GL-3, Invitrogen), anti-NK1.1 PE (clone:PK136, Biolegend), anti-Eomes APC (clone: Dan11mag, Invitrogen), and anti-CXCR3 BV750 (clone: CXCR3-173, BD Biosciences). Intracellular staining for Eomes was performed as previously described (35). To identify neutrophils (CD45^+^Ly6G^+^CD11b^+^) and macrophages (CD45^+^Ly6G^-^ Siglec-F^-^MHCII^+^), the gating strategy from Misharin et al. and the following antibodies were utilized; anti-CD45 AF 700 (clone: 30-F11, Biolegend), anti-MCHCII VF450 (clone:M5/114.15.2, Tonbo Biosciences), anti-CD11b PE (clone: M1/70, Tonbo Biosciences), anti-CD11c BV605 (clone: N418, Biolegend), anti-CD69 BV711 (clone: H1.2F3, Biolegend), anti-Ly6G FITC (clone: RB6-8C5, Tonbo Biosciences), and anti-Siglec F BV650 (clone: E50-2440, BD Biosciences). The acquisition of the data was performed using the Quanteon (Agilent) and analysis was performed with NovoExpress (Agilent) following the gating strategies in **Supplementary Figures 2** and **3** and Misharin et al. (36)

### Preparation of histopathological samples

Twelve, 5-day-old, female, C57BL/6J mice were randomly divided into 3 groups of 4 mice each. Mice received PBS (control) or 10^4^ CFU WT *B. pertussis* or *B. pertussisΔptx* in 15 μl PBS intranasally one time. Three days after inoculation, the animals were sacrificed with CO_2_ euthanasia. The lungs were infused with neutral-buffered, 10% formalin fixative solution and immersed in the same fixative. One week following necropsy, tissues were removed from formalin solution, immersed in 70% ethanol, and remained in alcohol until ready for processing. Tissues were subsequently processed, embedded in paraffin, sectioned at approximately 5 μm, and stained with hematoxylin and eosin (HE). A board-certified pathologist (UBM) performed all microscopic evaluations of the HE stained sections.

Microscopic exam consisted of evaluation of the lung for the presence or absence of inflammation. Microscopically, lesion (tissue change or alteration) incidence, severity, and distribution were recorded. If absent (i.e., histologically normal), a score of 0 was assigned. If present, the severity of the lesions was recorded as minimal, mild, moderate, or severe, with severity scores of 1 through 4, respectively, based on an increasing extent and/or complexity of change, unless otherwise specified. Lesion distribution was recorded as focal, multifocal, or diffuse, with distribution scores of 1, 2, or 3, respectively. The Group Histopathological Score was calculated by adding individual animal severity + distribution scores.

### Cytokine ELISA

Supernatant was taken from lung samples utilized for flow cytometry experiments. Samples were collected and stored at -20°C for cytokine analysis. 100 μl of supernatant from each lung sample was assessed for concentrations of IL-4 and IL-17 utilizing R&D Systems DuoSet ELISA kits following manufacturer’s instructions.

### Statistics

For experiments determining differences in bacterial loads in the organs of mice, the following statistical analyses were performed using GraphPad PRISM (GraphPad Software, Inc): Two-tailed unpaired Student t-tests, One-way ANOVA, and Two-way ANOVA. For experiments determining differences in immune cell populations, the following statistical analyses were performed using GraphPad PRISM (GraphPad Software, Inc): Two-tailed unpaired Student t-tests, One-way ANOVA, and Two-way ANOVA.

### Ethics statement

This study was carried out in accordance with the recommendations in the Guide for the Care and Use of Laboratory Animals of the National Institutes of Health. The protocol was approved by the Institutional Animal Care and Use Committees at The University of Georgia at Athens, GA (A2022 04-001-Y1-A0 Bordetella-Host Interactions, A2022 04-025-Y1-A0 Breeding Protocol, and A2022 04-022-Y1-A0 Neonatal Models of Bordetella infection, transmission, and immunity). Mice were consistently monitored for signs of distress over the course of the experiments to be removed from the experiment and euthanized using carbon dioxide inhalation to prevent unnecessary suffering.

## Results

### Neonatal T cell populations and susceptibility to *B. pertussis* transition in the first 2 weeks of life

**Figure 1:**
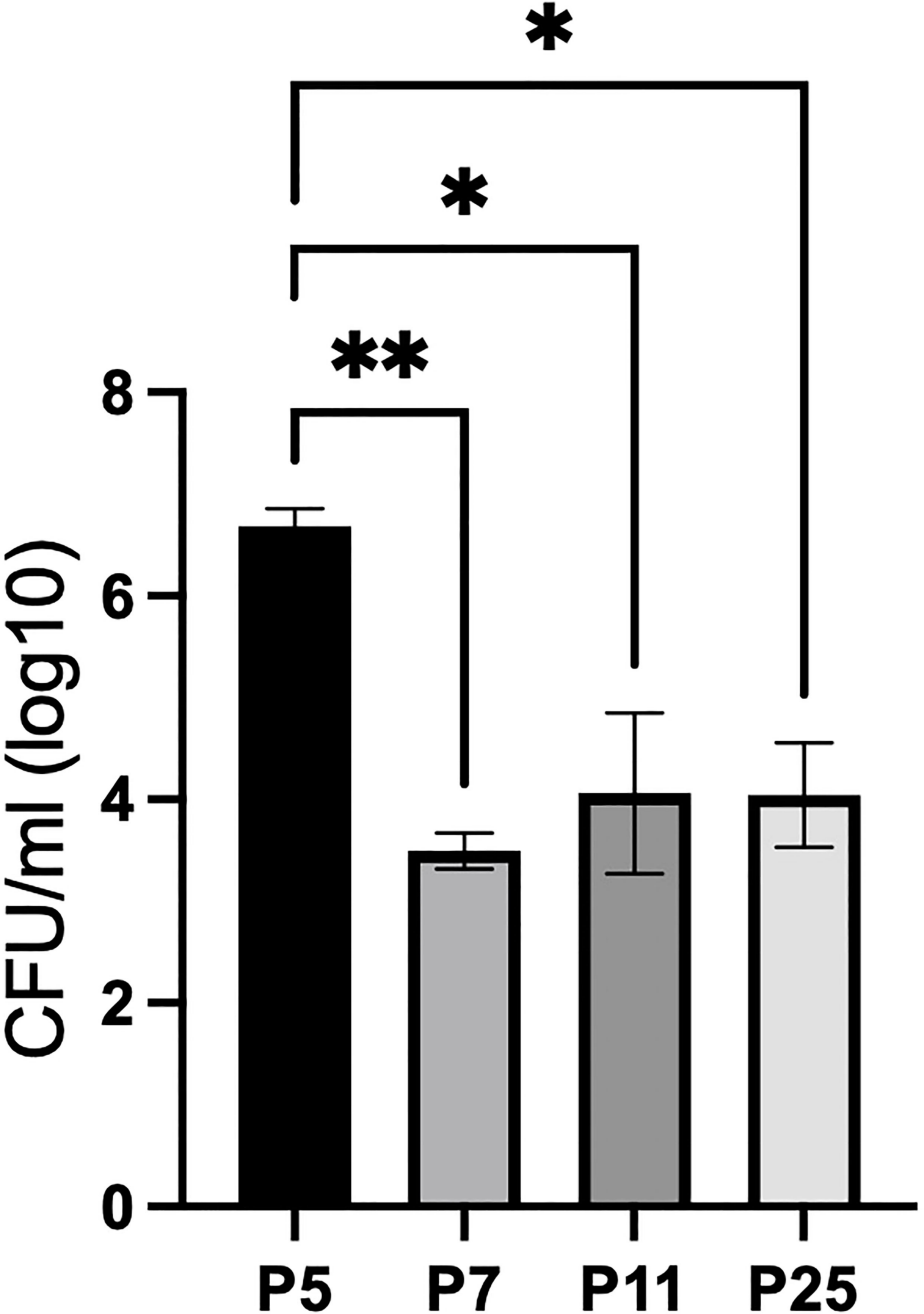
P5 C57BL/6J pups are significantly more susceptible to WT *Bp* than P7, P11, and P25 mice at 3 dpi (lungs). Mice were intranasally inoculated with 10^4^ CFU in 15 μl (P5, P7, and P11) or 50 μl (P25). Error bars show standard error of the mean, n=4. Statistical significance was calculated *via* One-way ANOVA. *p<0.0332, **p<0.0021.

To identify the appropriate aged mouse to assess neonatal susceptibility to *B. pertussis* (*Bp*), C57BL/6J mouse pups were inoculated with 10^4^ CFU *Bp* at five-, seven-, eleven-, and twenty-five-days-post birth (P5, P7, P11, and P25). Those inoculated at P7, P11, and P25 had roughly 10,000 CFU in their lungs 3 days post inoculation (dpi) (**Figure 1**), demonstrating growth similar to that observed in adult mice (**Supplementary Figure 1**). In contrast, mice inoculated at 5-days-old (P5) had approximately 5,000,000 CFU of *Bp* at 3 dpi (**Figure 1**), over 500-fold higher numbers than any older mice, indicating that P5 pups are significantly more susceptible to this neonatal pathogen than mice even 2 days older (P7). These results suggest there is a significant shift in immunological development distinguishing mice inoculated on P5 and P7.

To assess shifts in the pulmonary immune response that distinguishes P5-P8 mice from more mature mice, populations of B and T cells were assessed in the lungs of C57BL/6J mice at various ages. We observed significantly higher numbers of CD3^+^ T cells and CD19^+^ B cells in the lungs of P8 than P10, P14, and P28 mice, suggesting that despite high numbers of lymphocytes in the periphery, P5 mice are still highly susceptible to *Bp* (**Supplemental Figure 2**). Thus, it is likely that some feature of these lymphocytes in P5-P8 pups differ from those in P7, P10, P14, and P28 mice which results in significant susceptibility to *Bp.*

**Figure 2:**
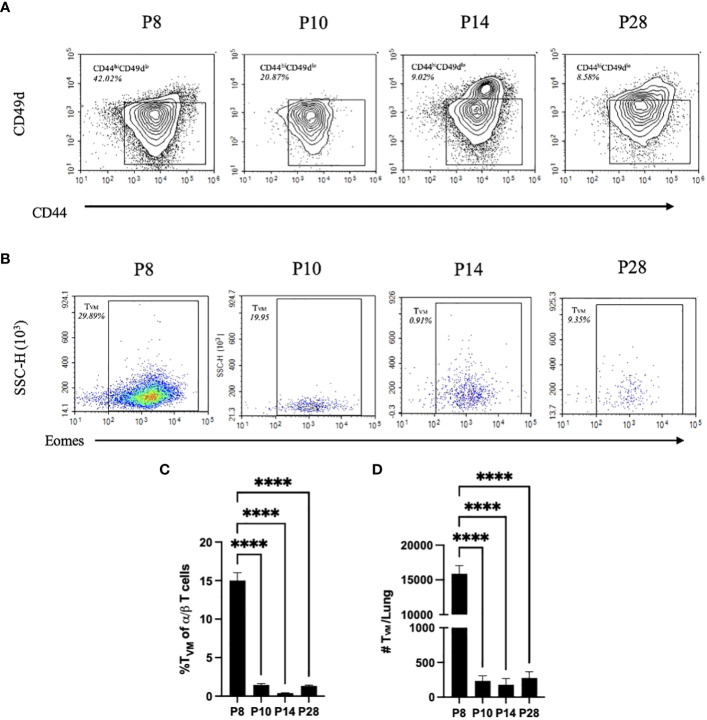
P10, P14, and P28 mice have significantly less T_VM_ than P8 mice. CD44^hi^CD49d^lo^ T cell populations from the lungs *via* gating strategy in **Supplementary Figure 3**. Proportion represented is frequency of CD44^hi^CD49d^lo^ T cells in a population of CD4/CD8 single positive α/β T cells **(A)**. Eomes expression from CD49d^lo^CD44^hi^CXCR3^+^ populations. Proportion represented is frequency of T_VM_ (CD49d^lo^CD44^hi^CXCR3^+^Eomes^+^) in a population of CD44^hi^CD49d^lo^ T cells **(B)**. Proportions of T_VM_ of CD4/CD8 single positive α/β T cells in the lungs from naïve P10, P14, and P28 mice **(C)**. Numbers of T_VM_ in the lungs from naïve P10, P14, and P28 mice **(D)**. (n=4 per group). Error bars show standard error of the mean. Statistical significance was calculated *via* One-way ANOVA. ns p> 0.0332 ****p<0.0001.

A key feature of immunological development is the maturation of the thymus and the T cells which develop therein. Therefore, subsets of T cells were characterized in the lung of P8, P10, P14, and P28 mice. A comprehensive flow cytometry panel was designed to assess shifts in populations of virtual memory T cells (T_VM_). Using the gating strategy in **Supplemental Figure 3**, we first identified populations of single positive CD3 α/β T cells *via* expression of CD3^+^NK1.1^-^TCRγδ^-^ and CD4^+^ or CD8^+^, then T_VM_ were identified *via* CD44^+^CD49d^-^CXCR3^+^Eomes^+^ expression. The panel and gating strategy were validated for specific binding *via* positive and negative controls (**Supplemental Figure 4**). Shifts in populations of CD44^hi^CD49d^-^ to CD44^hi^CD49d^+^ antigen experienced T cells were increasingly observed from P8 and P10 to P14 and P28 mice (**Figure 2A**). The decreased expression of Eomes and CXCR3 in these CD44^+^CD49d^-^ populations followed a similar trend as mouse age progressed, indicating that the highest proportions of T_VM_ were observed in P8 mice (**Figure 2B**). T_VM_ comprised a significantly larger proportion of single-positive T cells (15%) in the lungs of P8 mice, while only comprising 1-4% of T cells in P10, P14, and P28 mice (**Figure 2C**). This trend was also reflected in the total T_VM_ numbers in the lungs of P8 mice, which had ~75x greater numbers than P10, P14, or P28 mice (**Figure 2D**). Conversely, naïve adult T cells (CD44^lo^CD49d^lo^) made up only ~8% of single-positive T cells in the lungs of P8 mice, whereas they composed ~38% of single-positive T cells in the lungs of P10 mice (**Supplemental Figure 5**).

Since there is no objective cutoff distinguishing “neonatal” mice from later stages of development, we here use the dramatically different sensitivity to *Bp*, predominance of T_VM,_ and relative lack of naive T_adult_, as the basis for distinguishing P5-P8 mice, herein referred to as “neonatal”, from older mice referred to as “juvenile” (P10 to P21) or “adolescent” (P22 to P30) (**Figure 2**). Utilizing this framework, these data demonstrate that neonatal mice are much more susceptible to *Bp* and that increased populations of T_adult_ observed in P10 mice are associated with control of *Bp.*

### Pertussis toxin disrupts neonatal control of *B. pertussis*

**Figure 3:**
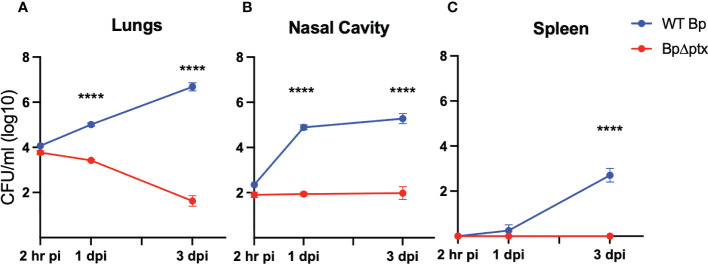
Pertussis toxin (PTx) is required for infection of P5 neonatal mice. Log10 of CFU recovered from the lungs **(A)** nasal cavity **(B)** and spleen **(C)** at 2 hours, 1, and 3 days post inoculation from neonatal C57BL/6J mice inoculated at P5 with either WT *B*. *pertussis* (blue) or *B*. *pertussisΔptx* (red) (n=4 per strain per timepoint). Statistical significance was calculated *via* Two-way ANOVA. Error bars show the standard error of the mean. ns p> 0.0332, ****p<0.0001.

To better understand the extraordinary susceptibility of five-day-old (P5) mice to *Bp*, we inoculated them as above and followed the progression over time in the respiratory tract as well as the spleen, which is colonized in severe infections. At 2 hours post inoculation, most of the initial inoculum was deposited in the lungs, confirming successful inoculation (**Figure 3A**). Within 3 days, *Bp* grew in the lungs over 100-fold to levels exceeding 1,000,000 CFU, indicating a failure of these neonates to control infection. *Bp* grew similarly unrestrained in the nasal cavities, from ~100 CFU at 2 hours post inoculation to over 100,000 CFU by 3 dpi (**Figure 3B**). The extraordinary susceptibility of P5 mice was further underscored by the sporadic splenic colonization on 1 dpi that grew to nearly 1,000 CFU in all mice by 3 dpi, indicating consistent systemic dissemination (**Figure 3C**), reflecting a serious pneumonic infection and failure of systemic immune control. These results demonstrate that when delivered to neonatal mice (P5), a relatively low dose of *Bp* can efficiently and consistently grow rapidly in the nose and lungs and disseminate to the spleen within 3 days post inoculation, characteristic of severe neonatal disease.

Multiple studies have identified pertussis toxin (PTx) as contributing to severe disease in juvenile (P14) and adult mice (22, 29, 32), but its effects have not been examined in neonatal (P5-P8) mice, as defined here. To assess the role of PTx in the exceptional virulence of *Bp* in our neonatal model, we compared the wild-type parental strain to an isogenic mutant with an in-frame deletion of the coding region of *ptx* (*BpΔptx*) (32). Inoculation with WT *Bp* and *BpΔptx* demonstrated similar recovery from the lungs at 2 hours post-inoculation, confirming equivalent delivery of both strains to the lungs (**Figure 3A**). Within 3 days, *BpΔptx* was nearly cleared from the lungs of neonatal mice, with only approximately 100 CFU remaining, while WT *Bp* grew nearly 1000x the original inoculation dose in this same period (**Figure 3A**). Despite consistent colonization of the spleen by WT *Bp*, there was no detected systemic dissemination to the spleen by *BpΔptx* (**Figure 3C**). Complementation of the *BpΔptx* mutant *via* co-inoculation with purified PTx resulted in partial recovery of the phenotype in the lungs (**Supplemental Figure 6**). This indicates that a single bolus delivery of PTx does not precisely mimic the continual secretion from the site of *Bp* microcolonies required for its effects. Together, these results indicate that the neonatal immune system can efficiently and rapidly control lung infection with the *BpΔptx* strain, but WT *Bp* substantially disrupts this ability *via* secretion of PTx.

Conventional infection models inoculate mice with supernaturally high doses of *Bp* (5x10^5^ CFU), potentially overcoming the most relevant host immune responses. Though C57BL/6 adult mice are approximately 10x the size of P5 pups, our much lower inoculation dose of 10^4^ CFU is 50x less than that delivered to adults. To examine the potential effects of high inocula in our neonatal mouse model, we used doses equivalent to those used in the conventional adult assays. Increasing the dose of *BpΔptx* delivered to P5 pups 10- and 100-fold, to 10^5^ and 10^6^ CFU, respectively, resulted in the death of most animals (**Supplemental Figure 7**). These results demonstrate that the neonatal immune system can be effective against moderate numbers resembling natural infection but can be overwhelmed by unnaturally large inocula. They also demonstrate that extremely high doses can disrupt the ability to study and understand the natural function of the host immune system.

### PTx disrupts rapid immune cell recruitment

**Figure 4:**
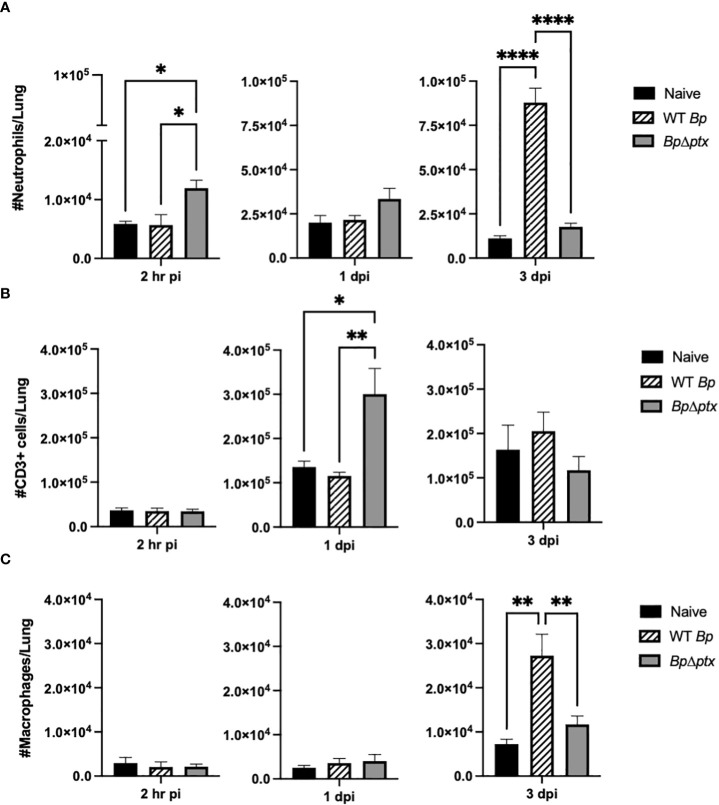
Pertussis toxin causes delayed neutrophil and T cell accumulation in the lungs of neonatal mice. Total neutrophils (CD45^+^Ly6G^+^CD11b^+^) in lungs of P5 C57BL/6J mice inoculated with WT *Bp*, *BpΔptx*, or uninfected at 2 hours, 1 day, and 3 days post inoculation **(A)**. Total T cells (CD3^+^) in lungs of P5 C57BL/6J mice inoculated with WT *Bp*, *BpΔptx*, or uninfected at 2 hours, 1 day, and 3 days post inoculation **(B)**. Total macrophages (CD45^+^Ly6G^-^Siglec-F^-^MHCII^+^) in lungs of P5 C57BL/6J mice inoculated with WT *Bp*, *BpΔptx*, or uninfected at 2 hours, 1 day, and 3 days post inoculation **(C)**. Statistical analysis was calculated *via* Two-way ANOVA. Error bars show standard error of the mean (n=5). *p<0.0332, **p<0.0021, and ****p< 0.0001.

PTx has been demonstrated to delay immune cell recruitment to the site of infection by approximately one week in adolescent and adult mice (22, 29). To quantify the effects of PTx on populations of immune cells recruited to neonatal lungs, P5 pups inoculated with WT *Bp* or *BpΔptx* were assessed *via* flow cytometry at 2 hours, 1 day, and 3 days post inoculation. At 2 hours post inoculation, pups inoculated with *BpΔptx* had significantly higher neutrophil counts in the lungs than pups inoculated with WT *Bp* (**Figure 4A**), indicating that PTx interferes with rapid neutrophil recruitment. By 1 dpi, *BpΔptx* was present in much lower numbers than WT *Bp* but had recruited significantly higher (3x more) numbers of CD3^+^ T cells into the lungs (**Figure 4B**), though numbers of T_VM_ in the lungs remained consistent across infection groups (**Supplemental Figure 8**). By 3 dpi, the mutant was nearly cleared from the lungs and the neutrophil and T cell numbers had already decreased, having substantially resolved the infection. In contrast, WT *Bp* had grown over 100-fold in number, but did not result in substantial increase in the populations of T cells in the lungs (**Figure 4B**). Instead, WT *Bp* resulted in significantly higher numbers of macrophages and neutrophils in lungs at 3 dpi, but these levels were not sufficient to clear or control the infection, as the bacterial load was 100-fold higher than the original inoculum and had already disseminated to the spleen (**Figures 4A, C**).

**Figure 5:**
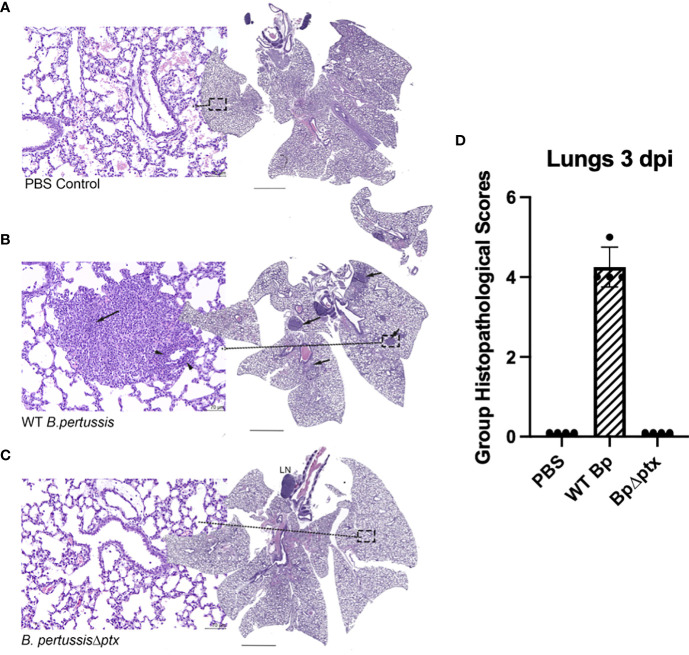
Pertussis toxin affects inflammation in the lungs of neonatal C57BL/6J mice. Representative images from neonatal lung mice exposed to PBS, WT *Bp*, or *BpΔptx* at 3 dpi. **(A)** PBS treated control. The center image is a whole-slide image from the lung. HE stain. Scale bar = 1.2 μm. The left image represents higher magnification (dashed box) of the lung. There are no significant tissue alterations. HE stain. Scale bar = 70 μm. **(B)** *Middle panel*: WT *B*. *pertussis*. The center image is a whole-slide image from the lung, with multiple hypercellular foci (arrows). HE stain. Scale bar = 1.2 μm. The left image represents higher magnification (dashed box) of the lung, with large number of neutrophilic and macrophage infiltrates obscuring the alveoli (arrow), and mild number of lymphocytes expanding the perivascular tissues (arrow heads). HE stain. Scale bar = 70 μm. **(C)** *B*. *pertussisΔptx*. The center image is a whole-slide image from the lung, with a distinct tracheobronchial lymph node (LN). HE stain. Scale bar = 1.2 μm. The left image represents higher magnification (dashed box) of the lung. There are no significant tissue alterations and resemble the lung tissues from PBS control mice. HE stain. Scale bar = 70 μm. **(D)** *Group Histopathological Scores*. In the current figure, Group Histopathological Scores, which were calculated by adding individual animal severity + distribution scores, were highest for the WT *B*. *pertussis* treated group at 3 dpi. The scores for the *B*. *pertussisΔptx* and PBS treated control groups were similar (**Supplementary Table 1**).

Histopathology of the lungs of infected mice was assessed as a measure of disease severity. To assess resulting inflammatory lesions in the lungs, P5 mice inoculated as above with WT *Bp, BpΔptx*, or PBS were histologically assessed at 3 dpi. H&E staining of lung sections of infected or naïve pups revealed that infection with *BpΔptx* did not result in observable inflammatory lesions and was indistinguishable from the PBS control pups (**Figures 5A, C, D**, and **Supplemental Table 1**). In contrast, WT *Bp* induced substantial inflammation, as evidenced by the presence of inflammatory lesions in 100% of mice assessed (**Figures 5B, D** and **Supplemental Table 1**).

### Pertussis toxin disrupts early cytokine production

**Figure 6:**
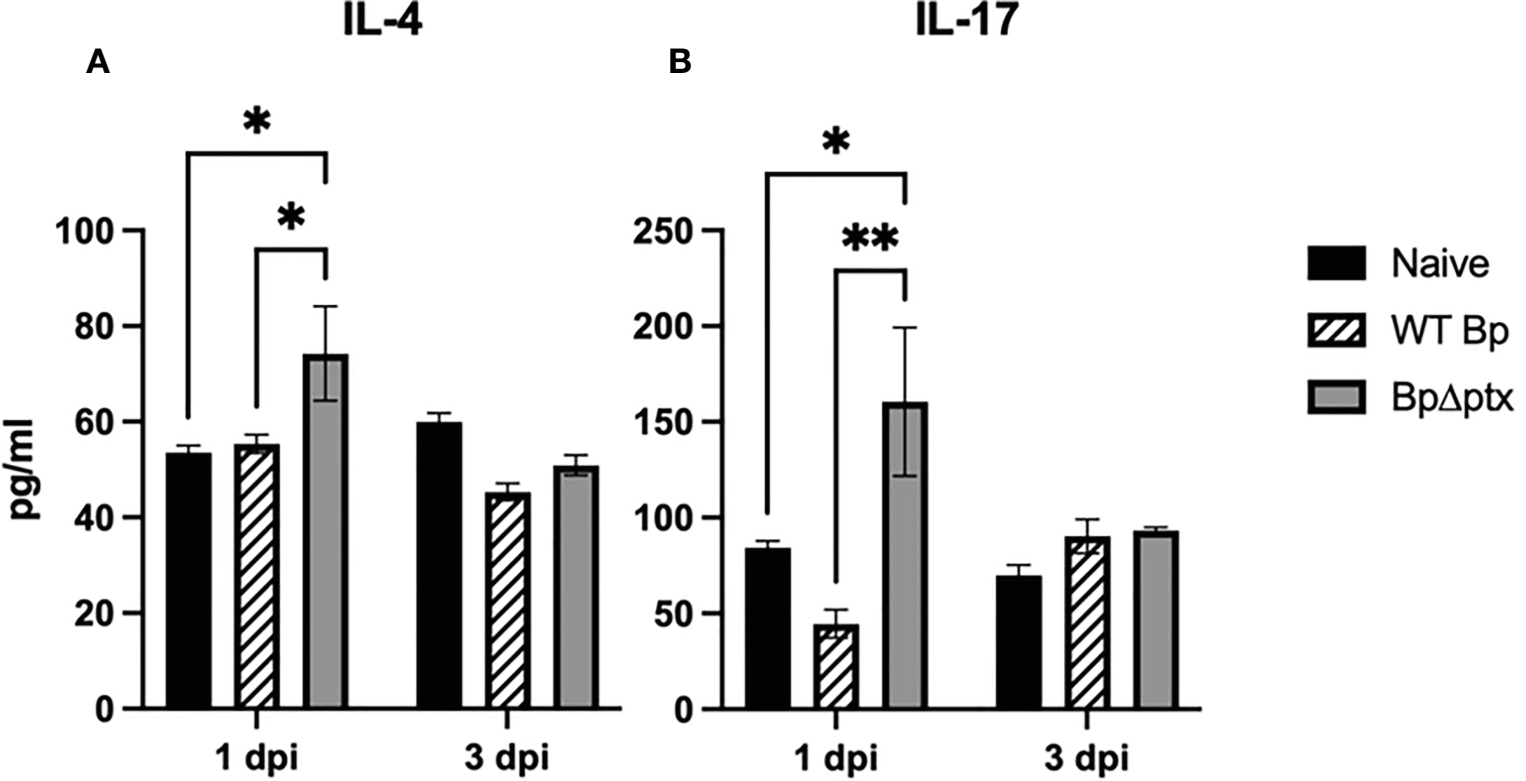
Pertussis toxin disrupts IL-4 and IL-17 production. Lung supernatant of C57BL/6J pups inoculated with WT *Bp*, *BpΔptx*, or uninfected and assessed at 1 dpi and 3 dpi. IL-4 pg/ml **(A)** and IL-17 pg/ml **(B)**. Statistical significance was calculated *via* Two-way ANOVA, n=5. Error bars represent standard error of the mean. *p<0.0332, **p<0.0021.

Neonatal immune responses are largely anti-inflammatory, which minimizes collateral tissue damage that might result from uncontrolled inflammatory responses to the onslaught of mostly harmless bacteria encountered at birth. We therefore assessed concentrations of representative anti-inflammatory (IL-4) and pro-inflammatory (IL-17) cytokines in neonates infected with either WT *Bp* or *BpΔptx.* At 1 dpi, pups inoculated at P5 with *BpΔptx* had significantly higher concentrations of both IL-4 and IL-17 isolated from lung supernatant than naïve and WT *Bp-*inoculated pups (**Figures 6A, B**). By 3 dpi, when the infection was nearly controlled, the concentrations of these cytokines in pups inoculated with *BpΔptx* returned to levels similar to the naïve control (**Figures 6A, B**). Surprisingly, pups inoculated with WT *Bp* did not have increased IL-4 or IL-17 in lung supernatants at 1 or 3 dpi, despite very high numbers of bacteria, significant immune cell recruitment, and formation of inflammatory lesions. These results suggest that PTx plays a pivotal role in suppressing the production of IL-4 and IL-17 in neonatal mice, thereby allowing the pathogen to grow to high numbers.

## Discussion

The increased mortality of neonates to infectious diseases has long been attributed to an inability to generate effective immune responses; however, this outlook fails to put into context the unique challenges faced by neonates and the immunological mechanisms employed to meet them (37). Immediately after birth, the naïve neonatal immune system is suddenly and continually thereafter exposed to innumerable microbes, both pathogenic and commensal. A critical requirement of the newborn’s immune system is that it avoids dangerously strong inflammatory responses to many harmless pathogens. For this reason, the neonatal immune response must be skewed towards anti-inflammatory cells and cytokines (6, 38–41). However, neonates must also rapidly respond to bacterial infections of critical and sensitive organs like the lungs and possess a set of unique immune cells which can rapidly respond to danger signals. These include myeloid-derived suppressor cells, CD71^+^ erythroid cells, and T_VM_, the latter of which respond rapidly to cytokine signals and a broad range of antigens in a memory-like fashion (10, 13, 34, 42–45). We enumerated population changes in T_VM_, which are numerous in the neonatal periphery before being replaced by adult T cells (T_adult_), demonstrating a shift in these cell types in post-natal development concurrent with dramatic changes in sensitivity to *Bp*.

Here, we present a novel model which more completely examines the unique neonatal response to a common bacterial pathogen which distinguishes neonatal pups (P5-P8) from a range of older mice. P7-10 mice have substantial numbers of naïve T_adult_ in their lungs and behave similarly to older mice when challenged with *Bp*. In stark contrast, mice just two days younger, P5-8, have larger populations of T_VM_, substantially lack T_adult_ in the lungs, and are highly susceptible to *Bp.* P14 mice were also observed to have a larger population of CD49d^+^ T cells in the lungs, possibly indicating a substantial shift from neonatal to juvenile mice (46). This model presents a novel opportunity to more accurately assess the neonatal immune response in the relative absence of T_adult_ that rapidly emerge in juvenile (P10-P21) mice.

In addition to T_VM_, neonates are enriched with CD71^+^ erythroid suppressor cells, which have been found to compromise the neonatal response to *Bp via* suppression of the innate immune response (6). Despite this and other immunosuppressive cells, we demonstrate that P5-P8 neonatal mice very efficiently controlled and nearly eliminated a pertussis toxin-deficient mutant (*BpΔptx*) within 3 days post inoculation, indicating that the neonatal immune response can be very effective against bacteria introduced into the lungs, but is highly sensitive to the effects of PTx. The rapid control of *BpΔptx* was associated with early accumulation of neutrophils and T cells to the lungs and early increases in IL-4 and IL-17 concentrations from lung supernatant. This model demonstrates a more profound effect of PTx on these neonatal mice that lack T_adult_ compared to juvenile and adult mice which are replete with T_adult_ (22, 47).

PTx is an extensively studied toxin with known effects on several aspects of the adult response to *Bp;* however, its principal role in pathogenesis remains to be determined. Though juvenile mice showed a somewhat larger effect, PTx has a modest effect in adolescent and adult mice compared to the >1,000-fold effect we observe here (22, 25, 32, 48–51). The largest effect of PTx previously observed was a ~10,000-fold reduction in rapid antibody-mediated bacterial clearance (29) In these experiments, transferred antibodies very rapidly cleared *BpΔptx* but not WT *Bp* from the lungs of adult mice, indicating that PTx has a profound effect on blocking rapid antibody-mediated clearance (29). We observe much faster control of *BpΔptx* in neonatal mice without the aid of transferred antibodies and observed rapid accumulation of neutrophils and T cells. These smaller numbers of *BpΔptx* resulted in less pathology in the lungs, while large numbers of WT *Bp* lead to more inflammatory lesions. Rather than a direct role in inducing pathology, it seems more likely that PTx blocks early immune responses critical to controlling bacterial infection, thus contributing to increased pathology. Altogether, PTx appears to block neonate-specific immune functions that could otherwise very efficiently eliminate *Bp*, allowing it to grow to large numbers and induce greater pathology.

The failure of current *Bp* vaccines to prevent transmission and nasal colonization among infants demonstrates limitations of the previous models used to develop these vaccines and emphasizes the necessity of a better understanding of host interactions, especially in highly sensitive newborns (20, 21, 52, 53). PTx appears to disrupt critical early responses unique to neonates, potentially explaining the specific sensitivity of neonates to *Bp*. Similar mechanisms may also be used by other pathogens to thwart the neonatal immune system and cause serious disease in this susceptible population (1, 2). Understanding the key functions that are blocked by PTx may reveal novel aspects of neonatal immunity that can guide efforts to protect this vulnerable population. This novel infection model may be useful to better understand how the neonatal immune system can be effective, in the absence of T_adult_, and why newborns are so susceptible to specific pathogens.

## Data availability statement

The raw data supporting the conclusions of this article will be made available by the authors, without undue reservation.

## Ethics statement

The animal study was reviewed and approved by Institutional Animal Care and Use Committees at the University of Georgia.

## Author contributions

CS, KD, NM, and EH conceived the study. CS, KD, and EH designed the experiments. CS, AC, MC, and UB-M performed the experiments. CS, AC, MC, and UB-M analyzed the data. CS and EH wrote the manuscript. All authors contributed to the article and approved the submitted version.

## Supplementary material

Supplementary Figure 1 Pertussis toxin has a modest effect on adult C57BL/6J mice at 3 dpi. Mice were intranasally inoculated with 10^4^ CFU of WT *B. pertussis* (*Bp*) (blue) or *B. pertussisΔptx* (Ptx) (red) in 50 μl PBS (lungs shown). Error bars show standard error of the mean, n=4. Statistical significance was calculated *via* student T test. ns>0.0332.

Supplementary Figure 2 P8 mice have increased lymphocyte populations. Total T cell populations (CD45^+^CD3^+^) from the lungs of naïve P8, P10, P14, and P28 C57BL/6 mice **(A)**. Total B cell populations (CD45^+^CD3^-^CD19^+^) from the lungs of naïve P8, P10, P14, and P28 C57BL/6J mice **(B)**. Gating strategy for lymphoid panel from lung of naïve P8 C57BL/6J mouse **(C)**. Error bars show standard error of the mean, n=4-5. Statistical significance was calculated *via* One-way ANOVA. ns p> 0.032, *p<0.0332, **p> 0.01.

Supplementary Figure 3 Gating strategy for the isolation of virtual memory T cells (T_VM_) from naïve P8 C57BL/6J pup lungs.

Supplementary Figure 4 Positive and negative controls for T_VM_ panel. Positive control was assessed *via* T_VM_ from P8 C57BL/6J pup lungs stimulated with PMA/ionomycin or unstimulated. Negative control was assessed *via* T_VM_ from P8 T cell^-/-^ pup lungs stimulated with PMA or unstimulated. Error bars show standard error of the mean, n=7-8. Statistical significance was calculated *via* Two-way ANOVA. ns p> 0.032 ***p<0.001.

Supplementary Figure 5 P8 C57BL/6J pups have significantly lower proportions of naïve T cells (CD3^+^Tcrγ/δ^-^CD4^+^CD8^+^CD44^lo^CD49d^lo^) than P10 C57BL/6J mice. Error bars show standard error of the mean, n=4. Statistical significance was calculated *via* One-way ANOVA. **p> 0.01 ****p<0.0001.

Supplementary Figure 6 Purified pertussis toxin (PTx) partially rescues ability of *BpΔptx* to cause disease in P5 neonatal mice. Log10 of CFU recovered from the lungs **(A)** and nasal cavity **(B)** from neonatal P5 C57BL/6J mice at 3 days post inoculation with WT *Bp* (blue), *BpΔptx* (red), or *BpΔptx* supplemented with purified PTx (*BpΔptx* + PTx) (purple) (n=4 per strain). Statistical analysis was calculated *via* Two-way ANOVA. Error bars show standard error of the mean. ns p> 0.0332, ***p< 0.0002, ****p< 0.0001.

Supplementary Figure 7 Effects of pertussis toxin on neonatal (P5-P8) mice are dose-dependent. Survival of mice inoculated at P5 with 10^5^ CFU of WT *Bp* or *BpΔptx* in 15 ul over 7 days **(A)**. Survival of mice inoculated at P5 with 10^6^ CFU of WT *Bp* or *BpΔptx* in 15 ul over 4 days **(B)**. Bacterial recovery from the lungs of mice inoculated with 10^5^ CFU of WT *Bp* or *BpΔptx* at 3 dpi **(C)** (dotted line indicates inoculation dose). Statistical analysis was calculated *via* student’s T test. ***p< 0.0002.

Supplementary Figure 8 T_VM_ populations in neonatal lungs are not affected by *B. pertussis*. Total number of T_VM_ in the lungs of pups inoculated with WT *Bp*, *BpΔptx*, or uninfected at 1 **(A)** and 3 **(B)** days post inoculation. T_VM_ are identified as CD3^+^NK1.1^-^TCRg/d^-^CD49d^lo^CD44^hi^CXCR3^+^Eomes^+^. Statistical analysis was calculated *via* One-way ANOVA. (n=4 per strain). Error bars show standard error of the mean. ns p> 0.0332.

Supplementary Table 1 Comparative group histopathological scores for the lungs of P5 C57BL/6J mice that were infected with WT *Bp*, *BpΔptx*, or PBS and assessed 3 dpi (n=4) (Sev= severity, Dis= distribution).
