# Supplementary figures and images for: Novel murine model reveals an early role for pertussis toxin in disrupting neonatal immunity to Bordetella pertussis
Source: Front Immunol. 2023 Feb 8;14:1125794. doi: 10.3389/fimmu.2023.1125794 (PMC9968397; doi:10.3389/fimmu.2023.1125794)

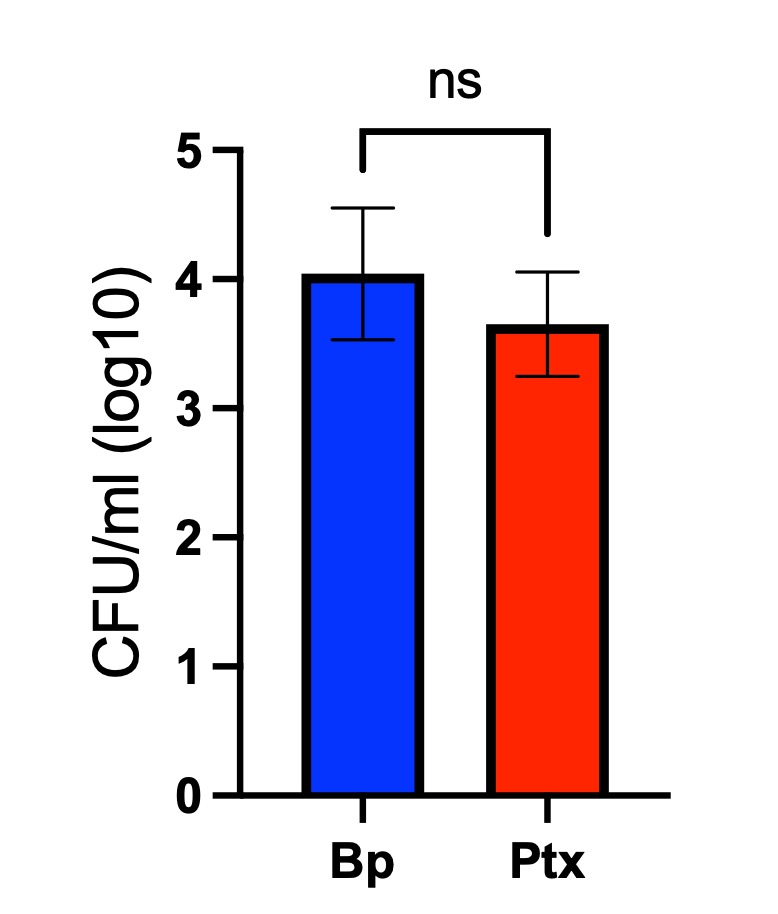

Supplement: Supplementary Figure 1 — Pertussis toxin has a modest effect on adult C57BL/6J mice at 3 dpi. Mice were intranasally inoculated with 104 CFU of WT B. pertussis (Bp) (blue) or B. pertussisΔptx (Ptx) (red) in 50 μl PBS (lungs shown). Error bars show standard error of the mean, n=4. Statistical significance was calculated via student T test. ns>0.0332. [file Image_1.jpeg]

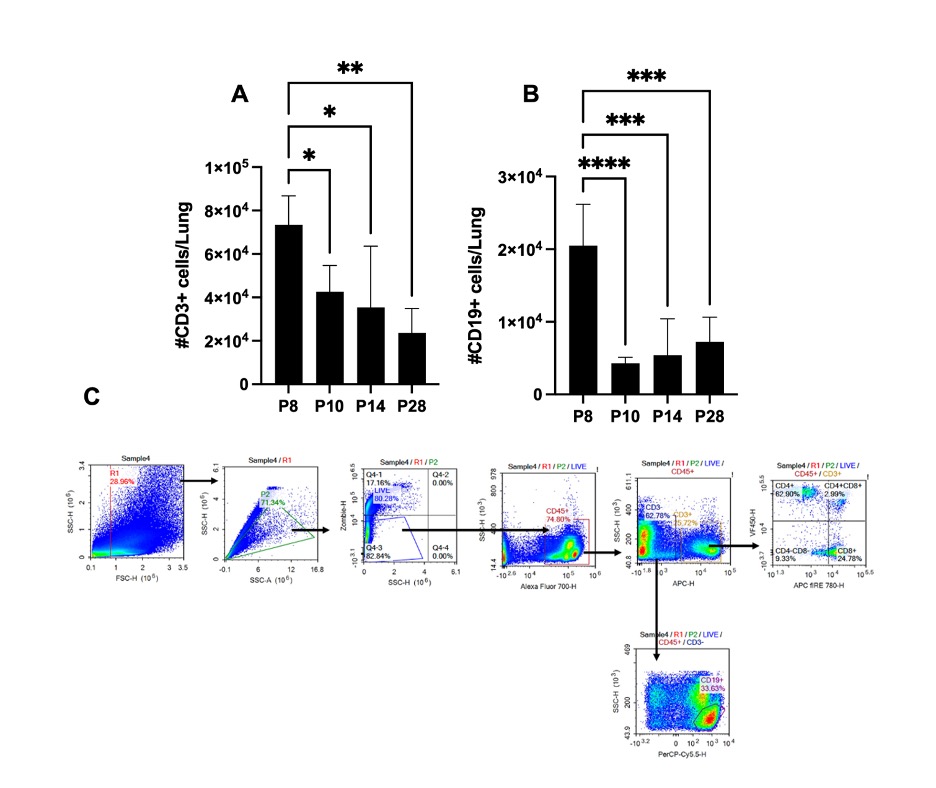

Supplement: Supplementary Figure 2 — P8 mice have increased lymphocyte populations. Total T cell populations (CD45+CD3+) from the lungs of naïve P8, P10, P14, and P28 C57BL/6 mice (A). Total B cell populations (CD45+CD3-CD19+) from the lungs of naïve P8, P10, P14, and P28 C57BL/6J mice (B). Gating strategy for lymphoid panel from lung of naïve P8 C57BL/6J mouse (C). Error bars show standard error of the mean, n=4-5. Statistical significance was calculated via One-way ANOVA. ns p> 0.032, *p<0.0332, **p> 0.01. [file Image_2.jpeg]

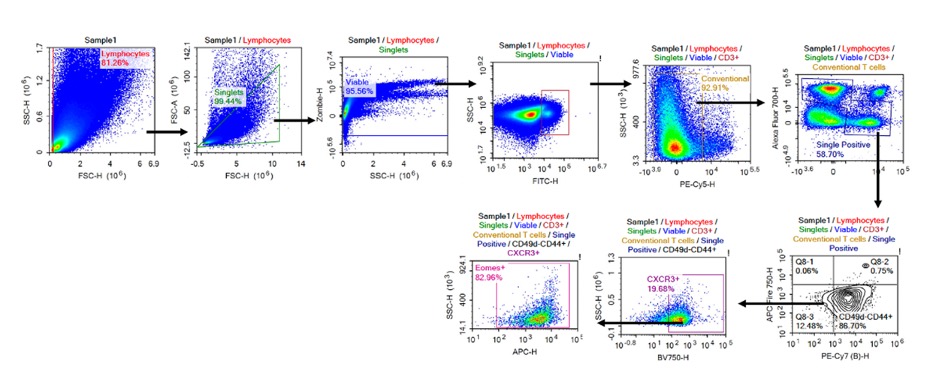

Supplement: Supplementary Figure 3 — Gating strategy for the isolation of virtual memory T cells (TVM) from naïve P8 C57BL/6J pup lungs. [file Image_3.jpeg]

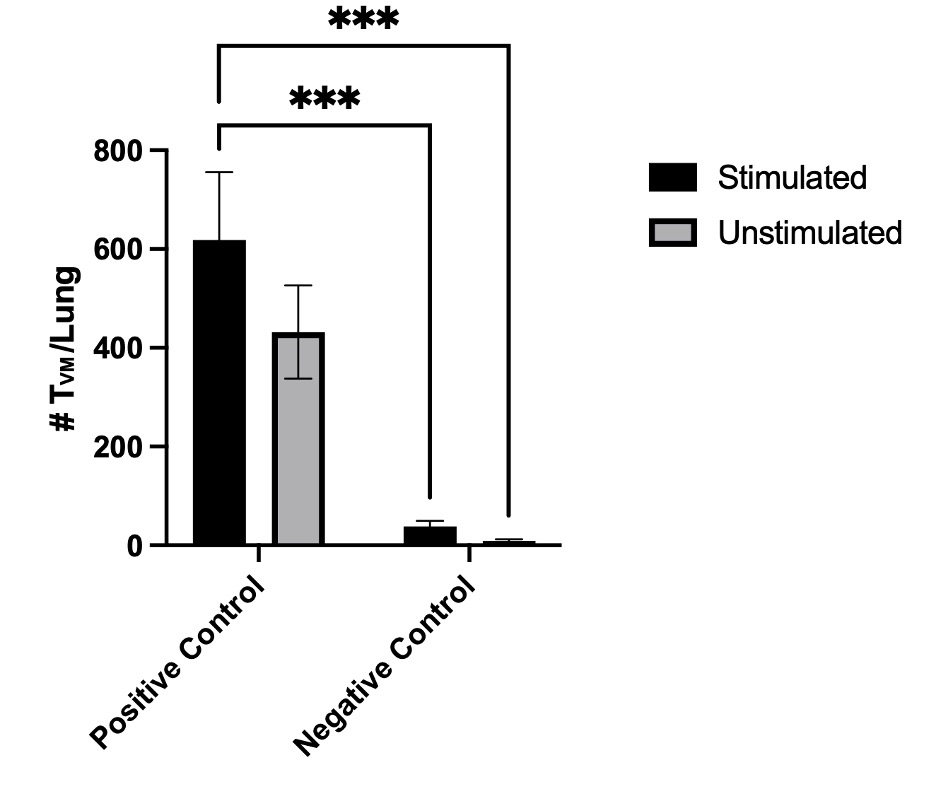

Supplement: Supplementary Figure 4 — Positive and negative controls for TVM panel. Positive control was assessed via TVM from P8 C57BL/6J pup lungs stimulated with PMA/ionomycin or unstimulated. Negative control was assessed via TVM from P8 T cell-/- pup lungs stimulated with PMA or unstimulated. Error bars show standard error of the mean, n=7-8. Statistical significance was calculated via Two-way ANOVA. ns p> 0.032 ***p<0.001. [file Image_4.jpeg]

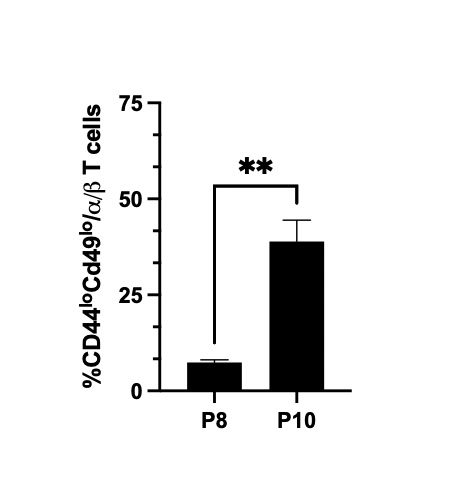

Supplement: Supplementary Figure 5 — P8 C57BL/6J pups have significantly lower proportions of naïve T cells (CD3+Tcrγ/δ-CD4+CD8+CD44loCD49dlo) than P10 C57BL/6J mice. Error bars show standard error of the mean, n=4. Statistical significance was calculated via One-way ANOVA. **p> 0.01 ****p<0.0001. [file Image_5.jpeg]

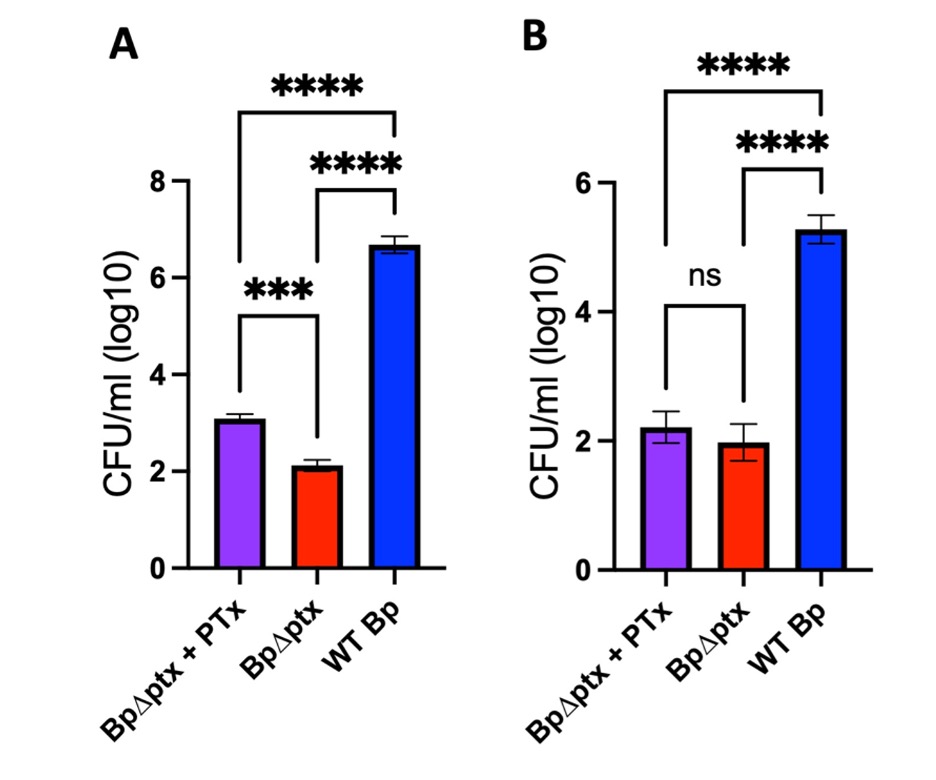

Supplement: Supplementary Figure 6 — Purified pertussis toxin (PTx) partially rescues ability of BpΔptx to cause disease in P5 neonatal mice. Log10 of CFU recovered from the lungs (A) and nasal cavity (B) from neonatal P5 C57BL/6J mice at 3 days post inoculation with WT Bp (blue), BpΔptx (red), or BpΔptx supplemented with purified PTx (BpΔptx + PTx) (purple) (n=4 per strain). Statistical analysis was calculated via Two-way ANOVA. Error bars show standard error of the mean. ns p> 0.0332, ***p< 0.0002, ****p< 0.0001. [file Image_6.jpeg]

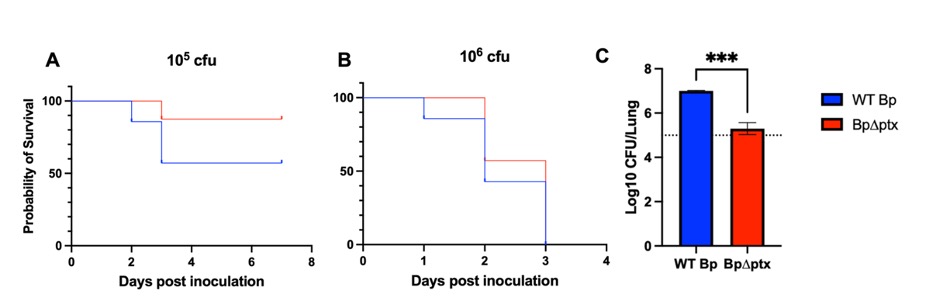

Supplement: Supplementary Figure 7 — Effects of pertussis toxin on neonatal (P5-P8) mice are dose-dependent. Survival of mice inoculated at P5 with 105 CFU of WT Bp or BpΔptx in 15 ul over 7 days (A). Survival of mice inoculated at P5 with 106 CFU of WT Bp or BpΔptx in 15 ul over 4 days (B). Bacterial recovery from the lungs of mice inoculated with 105 CFU of WT Bp or BpΔptx at 3 dpi (C) (dotted line indicates inoculation dose). Statistical analysis was calculated via student’s T test. ***p< 0.0002. [file Image_7.jpeg]

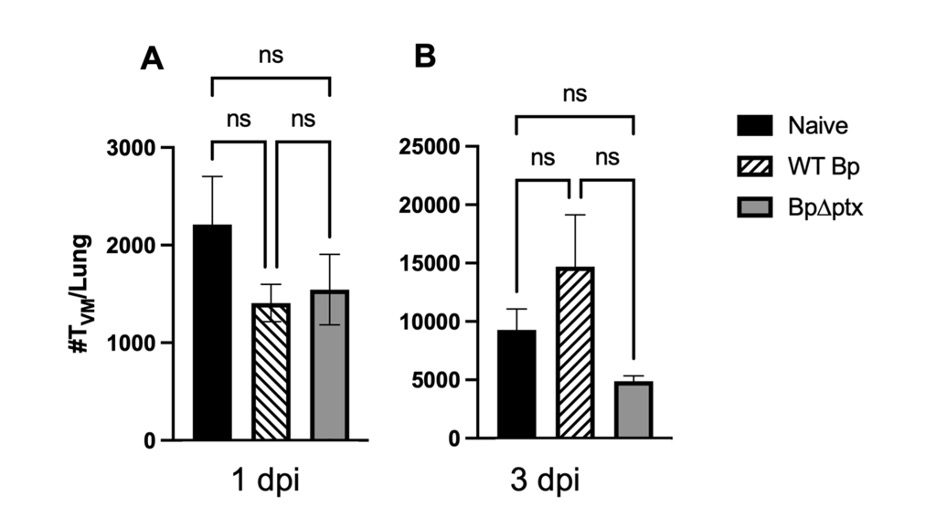

Supplement: Supplementary Figure 8 — TVM populations in neonatal lungs are not affected by B. pertussis. Total number of TVM in the lungs of pups inoculated with WT Bp, BpΔptx, or uninfected at 1 (A) and 3 (B) days post inoculation. TVM are identified as CD3+NK1.1-TCRg/d-CD49dloCD44hiCXCR3+Eomes+. Statistical analysis was calculated via One-way ANOVA. (n=4 per strain). Error bars show standard error of the mean. ns p> 0.0332. [file Image_8.jpeg]

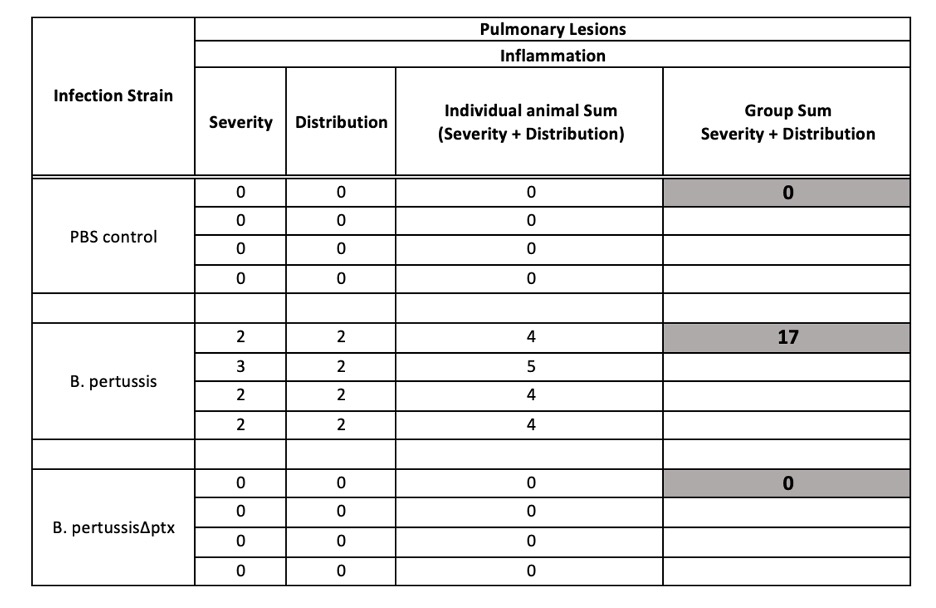

Supplement: Supplementary Table 1 — Comparative group histopathological scores for the lungs of P5 C57BL/6J mice that were infected with WT Bp, BpΔptx, or PBS and assessed 3 dpi (n=4) (Sev= severity, Dis= distribution). [file Image_9.jpeg]
